# A suspensory fixation technique for calcaneal tuberosity avulsion fractures using the TightRope Attachable Button System

**DOI:** 10.1016/j.tcr.2024.101096

**Published:** 2024-09-14

**Authors:** Gopikrishnan S. Nair, Nima Razii, Ting Y. Tan, Robert L. Carter, Stuart W. Bell

**Affiliations:** Department of Trauma and Orthopaedics, Queen Elizabeth University Hospital, Glasgow, United Kingdom

**Keywords:** Os calcis fracture, Calcaneal tuberosity, Beak fracture, Tongue-type fracture, Achilles tendon, Avulsion

## Abstract

Displaced avulsion fractures of the calcaneal tuberosity generally occur as a result of osteoporotic insufficiency or high-energy injuries. Conventional methods of fixation may be complicated by wound breakdown, metalwork failure, or symptomatic hardware. This is particularly relevant in elderly patients and those with comorbidities, including osteoporosis or diabetes. We describe an innovative technique using the TightRope Attachable Button System (ABS; Arthrex, Naples, FL, USA), adapted from suspensory cortical fixation in anterior cruciate ligament reconstruction, to treat displaced Beavis type II ‘beak’ calcaneal fractures in such patients. We present the case of a 67 year old female with multiple comorbidities, who successfully underwent this procedure, with no complications at 4 years follow-up.

## Introduction

The os calcis is the most commonly fractured tarsal bone, often in the context of high-energy injuries, although fewer than 40 % of calcaneal fractures are extra-articular [[1], [2], [3]]. Amongst these, calcaneal tuberosity avulsions only represent around 2 % of all calcaneal fractures [[4], [5], [6]], and are generally associated with osteoporotic insufficiency in elderly patients and those with comorbidities, such as diabetes [[7], [8], [9]]. Superior rotational displacement in the sagittal plane of the avulsed fragment from the strong pull of the triceps surae may result in skin necrosis due to pressure, or painful nonunion [2,4]. There is little evidence around nonoperative management, although it may be considered for fractures with minimal displacement, or patients with very significant comorbidities or anaesthetic risks [3,9].

**Fig. 1:**
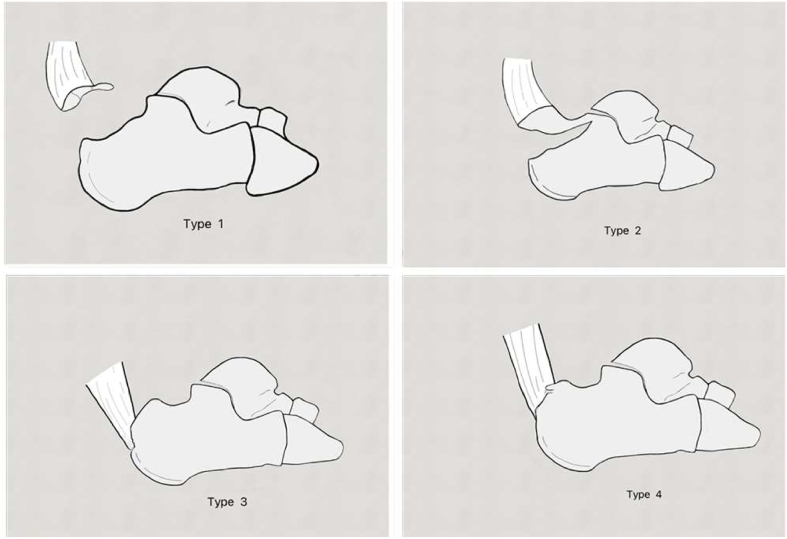
Lee classification of calcaneal tuberosity avulsion fractures (modification of the Beavis classification) [5,6]. (Reproduced from Liu et al. [9], with permission from Elsevier.)

Beavis et al. proposed a classification for these fractures, in which type I is a ‘sleeve’ fracture, type II is the classical ‘beak’ morphology, and type III is an infrabursal avulsion [5]. Lee et al. subsequently described a type IV fracture [6], with a much smaller ‘beak’ involving only the deep fibres of the Achilles tendon insertion (Fig. 1). Open reduction and internal fixation with lag screws has conventionally been the standard treatment, especially with type II fractures. However, lag screw fixation of such injuries may not overcome the pull out force of the Achilles tendon.

The Achilles tendon transmits forces of 2 to 3 times the body weight during normal walking, which increases to >6 times the body weight when running [10,11]. Avulsion fractures of the calcaneal tuberosity are typically caused by sudden contraction of the gastrocnemius-soleus complex whilst landing on the foot, particularly in osteoporotic bone, which explains why they tend to be more common in elderly females [2,12]. However, they have also been observed in younger patients who suffer higher-energy trauma, such as a direct impact to the hindfoot [6].

Complication rates following surgical fixation of calcaneal tuberosity avulsion fractures are reported between 30 % and 70 % in the literature, and predominantly comprise soft tissue complications (such as skin necrosis or wound breakdown), followed by loss of fixation [3,13,14]. The risk of complications is unsurprisingly higher in the presence of compromised soft tissues or poor quality bone [3]. We have developed a novel technique, adapted from suspensory cortical fixation in anterior cruciate ligament (ACL) reconstruction, to treat displaced Beavis type II fractures in such patients, as described in this report.

## Case presentation

**Fig. 2:**
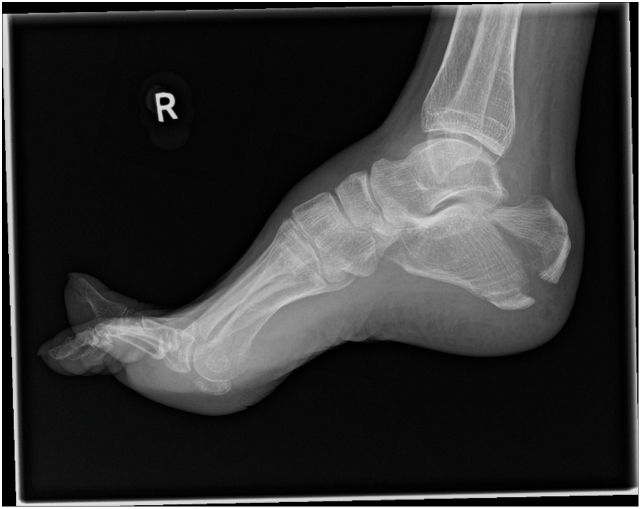
Lateral right foot radiograph demonstrates a displaced ‘beak’ calcaneal tuberosity avulsion fracture.

A 67 year old female fell down 5 concrete steps, sustaining a closed injury to her right os calcis. Her past medical history included severe chronic obstructive airways disease (requiring home nebulisers and frequent courses of oral corticosteroids for infective exacerbations), heavy smoking, and a low body mass index of 15, weighing 34.2 kg. She had no history of diabetes mellitus or previous podiatric issues. Examination revealed tenderness with bruising and swelling overlying the hindfoot, and a palpable fracture fragment underneath intact skin with good capillary refill, not considered to be imminently at risk of necrosis. Plain radiographs demonstrated a displaced Beavis type II ‘beak’ avulsion fracture of the calcaneal tuberosity (Fig. 2).

After discussing treatment options with the patient, she provided informed consent to undergoing surgical fixation the following day. Traditional methods of open reduction and internal fixation were deemed a significant risk to failure in view of the patient's comorbidities. We therefore elected to modify a technique initially described by Harb et al. [15], with the supplementation of an Attachable Button System (TightRope ABS; Arthrex, Naples, FL, USA) – a suspensory fixation device used in ACL reconstruction. The aim of the novel addition of the attachable button component to the construct was to provide a larger surface area for cortical bone fixation and maintain reduction of this fracture. In addition, the technique avoids an incision directly over the Achilles tendon, whilst ensuring that any plantar incision is minimally invasive.

**Fig. 3:**
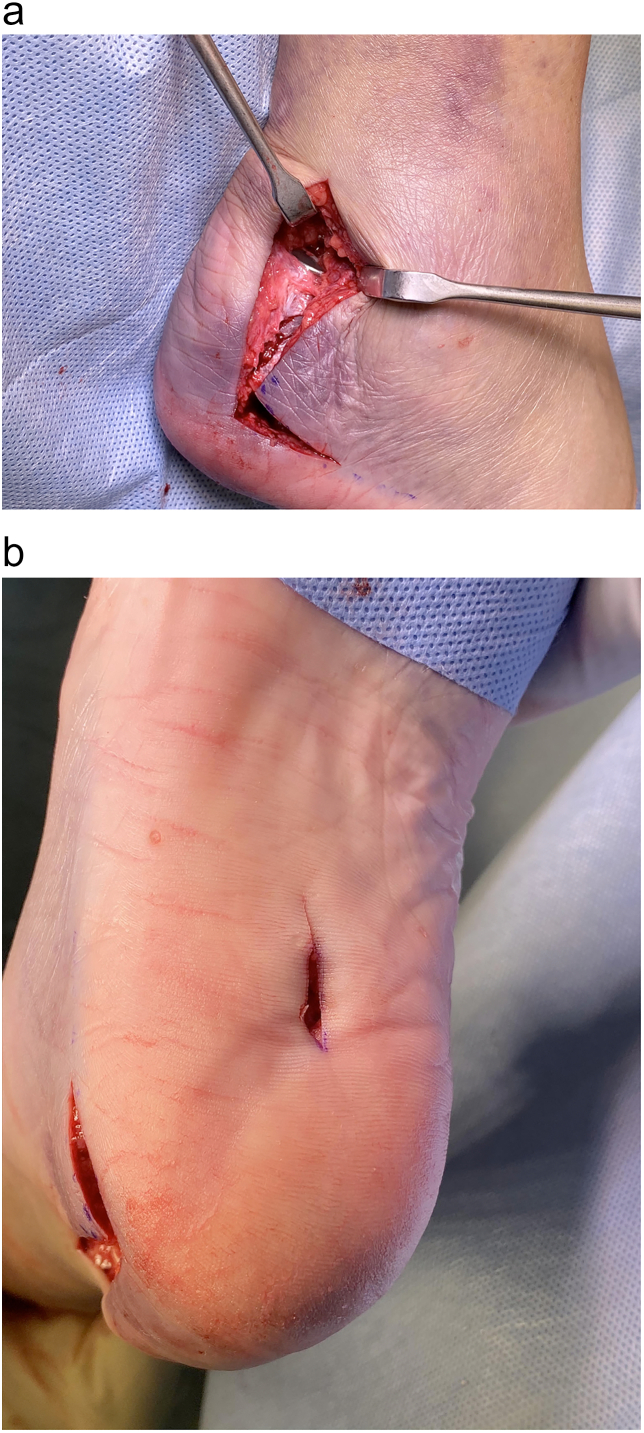
Reduction of the fracture was performed through an ‘L-shape’ extended lateral approach **(a)** to the hindfoot, taking a full-thickness flap to protect the sural nerve and preserve the blood supply to the skin, and a percutaneous stab incision **(b)** just beyond the weight-bearing surface of the heel. This method of fixation can also be performed through a smaller percutaneous incision, due to the precision of an ACL drill guide.

The patient underwent surgery in the lateral decubitus position with the knee slightly flexed to relax the triceps surae, following spinal anaesthesia and a sciatic nerve block. An above-knee tourniquet was inflated to 250 mmHg after standard antibiotic prophylaxis with IV cefuroxime was administered. An extended lateral approach to the hindfoot, as described by Freeman et al. [16], was performed through an ‘L-shape’ incision, taking a full-thickness flap to protect the sural nerve and preserve the blood supply to the skin (Fig. 3). Having identified the fracture through the lateral approach, which was clear of any soft tissue interposition, direct reduction was performed with a large fragment AO clamp. A percutaneous stab incision just anterior to the weight-bearing surface of the heel provided access to the plantar surface of the os calcis.

**Fig. 4:**
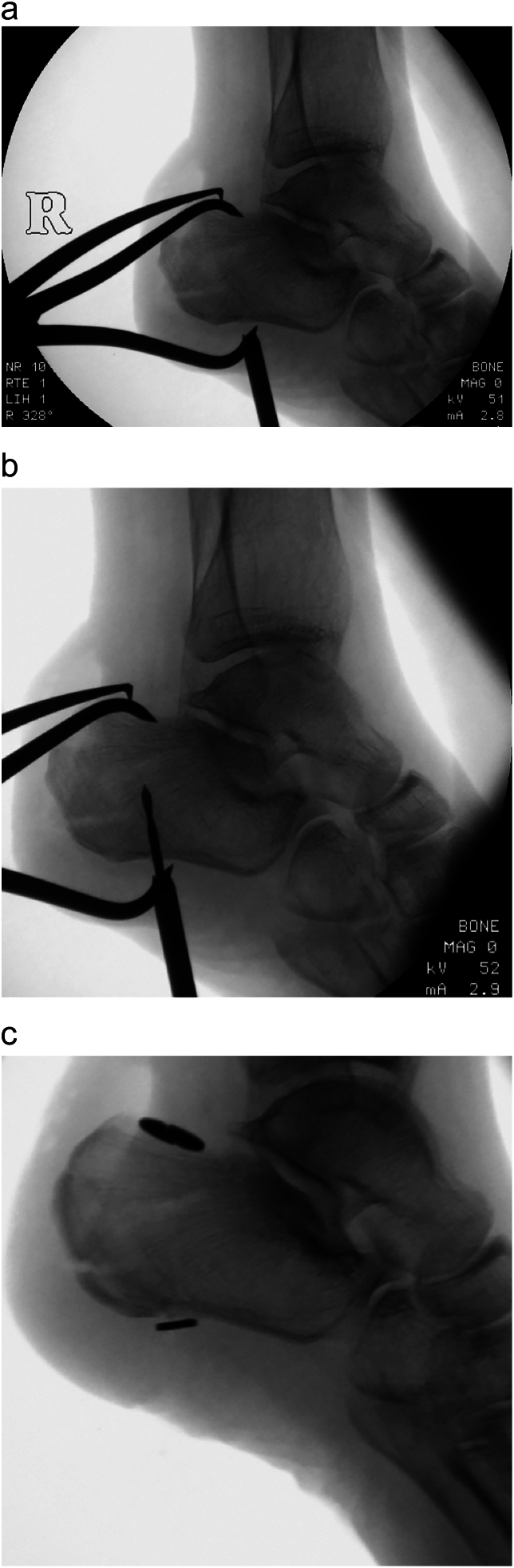
Whilst reduction is maintained with a large fragment AO clamp **(a)**, an ACL drill guide is used to pass a 2.4 mm guide wire retrograde through the fracture **(b)**, before the TightRope is secured with an ABS button on the dorsal side and tensioned with an RT device on the plantar side **(c)**.

**Fig. 5:**
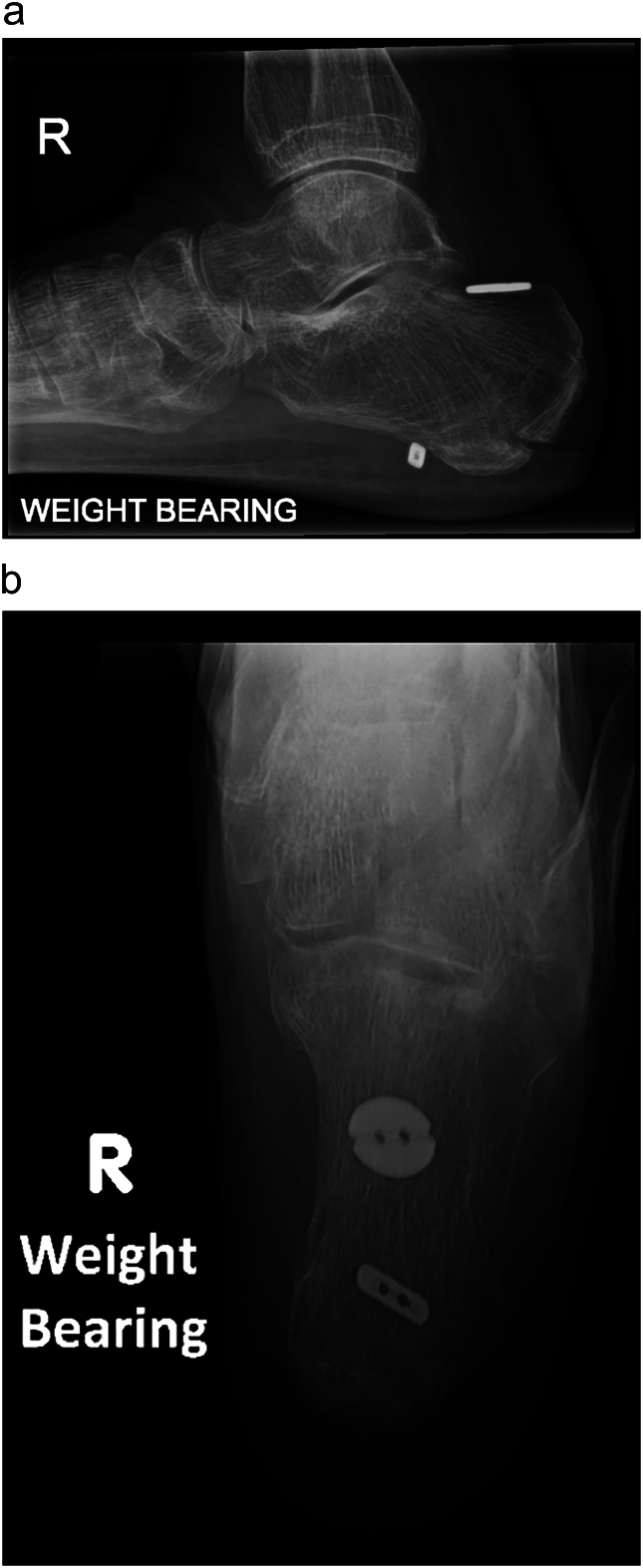
Lateral **(a)** and axial **(b)** right calcaneal radiographs at 3 months follow-up demonstrate fracture union, with no change in position of the fixation.

**Fig. 6:**
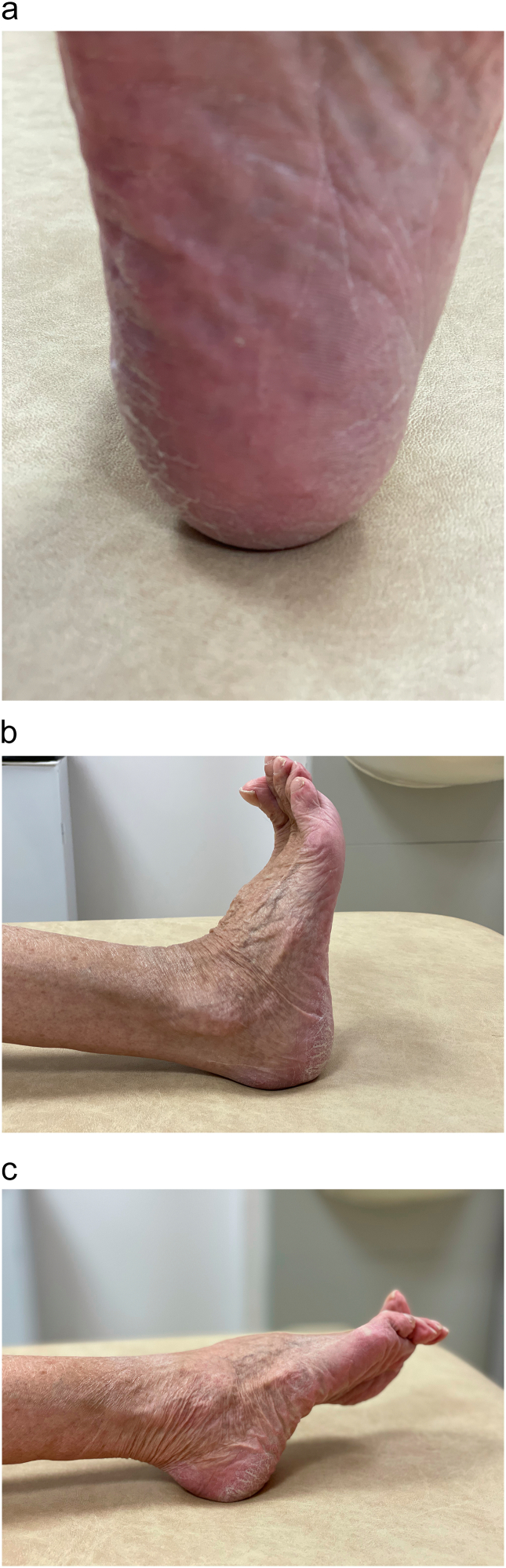
Clinical photographs at 1 year follow-up demonstrate well-healed surgical scars on the plantar **(a)** and lateral **(b)** aspects of the right hindfoot, with a full arc of ankle motion **(c)**.

After reduction was achieved, a tibial ACL drill guide was utilised to pass a 2.4 mm guide wire retrograde, perpendicular to the fracture (Fig. 4), towards the retrocalcaneal bursa. A 14 mm diameter round attachable button was secured dorsally, with an adjustable loop reverse tensioning device (TightRope RT; Arthrex) on the plantar aspect. This achieved good compression of the fracture fragment. The wound was closed in layers and the patient fitted with an equinus backslab, in which she was allowed to immediately mobilise toe-touch weight bearing with crutches. Full weight bearing in plantigrade was commenced at 6 weeks postoperatively. Check radiographs at 3 months follow-up demonstrated that the fracture had united (Fig. 5), and the patient was ambulating pain-free without requiring any walking aids. Clinical photographs were obtained at 1 year (Fig. 6), showing well-healed surgical scars and a full arc of ankle motion, which was maintained at most recent follow-up of 4 years.

## Discussion

This report describes a technique, which has been adapted from suspensory cortical button fixation in ACL reconstruction, to treat displaced ‘beak’ avulsion fractures of the calcaneal tuberosity. Any method of fixation for such injuries must withstand the forces transmitted through the Achilles tendon whilst the fracture unites. Traditional open reduction and internal fixation uses lag screws or wires inserted perpendicular to the fracture line, but since this tends to be in a similar direction of pull as the Achilles tendon, they are at particular risk of failure [12,17]. In a case series of 33 patients with calcaneal tuberosity avulsion fractures, Gitajn et al. identified a relatively high incidence of medical comorbidities (including smoking) amongst the entire cohort [3]. They also found that older patients and those with more than one comorbidity were significantly more likely suffer from postoperative complications. In a recent case series of 21 patients, Carnero-Martín de Soto et al. observed a complication rate of 70 % amongst 17 individuals who underwent surgery, with a fracture displacement of ≥20 mm being identified as a significant risk factor [14]. Whilst neither of these studies reported on rates of subsequent metalwork removal, it was required in almost a third of operated cases in a series of 58 intra-articular tongue-type fractures [18], which generally involve similar treatment techniques.

In view of the challenges associated with traditional methods of fixation, alternative techniques have been proposed, including transosseous suture fixation distally through bone tunnels in the os calcis, which can be reinforced with lag screws if the bony fragment is sufficiently large [19], and proximal sutures along the Achilles tendon using the Krackow technique. Transosseous suture fixation can be achieved using suture anchors, as described in several case reports [5,17,20,21]; alternatively, the suture ends can be passed through the plantar cortex of the calcaneal tuberosity just beyond the weight-bearing surface of the heel, and tied over the plantar fascia [12,22]. Greenhagen et al. describe excision of highly comminuted fracture fragments in a Charcot foot, with reattachment of the Achilles tendon to the remaining calcaneal tuberosity using a double row anchor system (SutureBridge; Arthrex) [23]. Based on encouraging results from tension band constructs [7,24], Agni and Fearon proposed the use of a locking compression hook plate through a lateral approach, which reduces the risk of peroneal irritation and symptomatic hardware due to its low profile [25].

Harb et al. adapted an ankle syndesmosis suture device for the fixation of a displaced type I fracture, in which a tunnel was drilled antegrade from the proximal fragment through the calcaneal tuberosity and a distal button secured against the non-weight bearing plantar cortex; meanwhile, the proximal FiberWire sutures (Arthrex) were passed through a Mayo needle and secured into the Achilles tendon [15]. In the present study, we avoided an incision directly over the Achilles tendon, and therefore drilled retrograde from the distal fragment using an ACL guide (Fig. 4). Instead of proximal fixation to the Achilles tendon, we used a round attachable button, which would provide a relatively large cortical footprint against the fracture fragment in the retrocalcaneal bursa, whilst fracture compression was achieved with the reverse-tensioning device on the plantar cortex. Although we used an extended lateral incision in order to directly visualise the reduction as a novel application of the ABS button (Fig. 3), this method of proximal suspensory fixation could easily be performed through a smaller percutaneous incision, due to the precision of an ACL drill guide.

Interestingly, suspensory fixation has been successfully adapted in the treatment of posterior cruciate ligament (PCL) avulsion fractures [26], which share some of the challenges posed by calcaneal tuberosity avulsion fractures, including the risk of cut-out and bony fragment destruction with conventional lag screw fixation, in addition to deforming forces from the PCL (as with the Achilles tendon). Likewise, we observed similar benefits to Wajsfisz et al. with the aid of a tip aiming drill guide for accurate placement of a single bony tunnel, since additional drill passes through the fracture fragment would have increased the risk of iatrogenic comminution.

In conclusion, there is no definitive consensus as to the most appropriate method of fixation for displaced calcaneal tuberosity avulsion fractures. As these are relatively uncommon injuries, the contemporary literature is typically presented in the form of similar case reports and technical notes. Although further research is needed, existing studies have collectively helped to identify useful learning points in the management of these challenging fractures. Indeed, it has been shown that they tend to occur in patients with factors associated with increased postoperative complications; therefore various innovative surgical techniques have been developed to reduce the risks of fixation failure.

## Funding

None.

## CRediT authorship contribution statement

**Gopikrishnan S. Nair:** Writing – review & editing, Writing – original draft, Visualization, Investigation. **Nima Razii:** Writing – review & editing, Writing – original draft, Visualization, Supervision, Investigation, Conceptualization. **Ting Y. Tan:** Writing – review & editing, Writing – original draft, Investigation. **Robert L. Carter:** Writing – review & editing, Writing – original draft, Supervision, Investigation, Conceptualization. **Stuart W. Bell:** Writing – review & editing, Writing – original draft, Visualization, Supervision, Investigation, Conceptualization.

## Declaration of competing interest

None.
